# Thermal Degradation of Polymers at High Temperatures*^1^

**DOI:** 10.6028/jres.063A.020

**Published:** 1959-12-01

**Authors:** Samuel L. Madorsky, Sidney Straus

## Abstract

Work on thermal degradation of polymers has previously been carried out at temperatures up to about 500° C. In the present work the range has been extended to 850° C. Polystyrene was pyrolyzed in a vacuum and also in helium at atmospheric pressure at 362° and at 850° C. Analysis of the volatile products indicates that higher temperatures and higher pressures cause a greater fragmentation of the volatile products. Samples of poly (vinylidene fluoride), polyacrylonitrile, and polytrivinylbenzene, were pyrolyzed in a vacuum at temperatures from 350° to 800° C. The more volatile products were analyzed qualitatively and quantitatively in a mass spectrometer. The less volatile products were tested for their average molecular weight by a microcryoscopic method.

Rates of thermal degradation were also determined for the last three polymers. The activation energies in the temperature range 218° to 440° C were found to be 48, 31, and 73 kcal/mole, respectively, for poly(vinylidene fluoride), polyacrylonitrile, and polytrivinylbenzene.

## 1. Introduction

This investigation was undertaken to study the thermal stability and products of degradation of polymers at temperatures up to 850° C. The polymers studied were polystyrene, poly (vinylidene fluoride), polyacrylonitrile, and polytrivinylbenzene.

Earlier work on thermal behavior of polymers at temperatures up to about 500° C has shown, among other things, that their stability and the mechanism of thermal degradation, as well as the chemical nature and relative amounts of the volatile and nonvolatile products of degradation, depend primarily on the molecular structure of the polymer. Thus, for example, tertiary carbon atoms in the chain weaken the adjacent C—C bonds in the chain. Quaternary carbon atoms are even more effective in this respect than tertiary carbons. Abundance of hydrogen on the chain leads to the formation, through random scissions, of volatile products which vary in size from those containing one carbon atom to those containing as many carbons as will permit the fragments to vaporize at the temperature employed in the pyrolysis. On the other hand, scarcity of hydrogen on the main chain will cause the formation of monomers by an unzipping process at the original chain ends as well as at the new ends formed by random scissions.

With regard to the nature of thermal degradation, several types of polymers should be differentiated:

1. Polymers in which scissions occur primarily in the backbone of the chain. These polymers tend to vaporize completely at sufficiently high temperatures.
2. Polymers in which the scissions occur primarily between the carbons of the backbone and the side groups. Such scissions result in formation of double bonds in the chain and, perhaps, also crosslinkages between the chains [1]^2^. On prolonged heating such polymers become more or less stabilized in the form of a partially carbonized residue.
3. Polymers that are highly crosslinked. These polymers are converted on heating into a honeycombed structure of carbonized residue.

**Figure 1:**
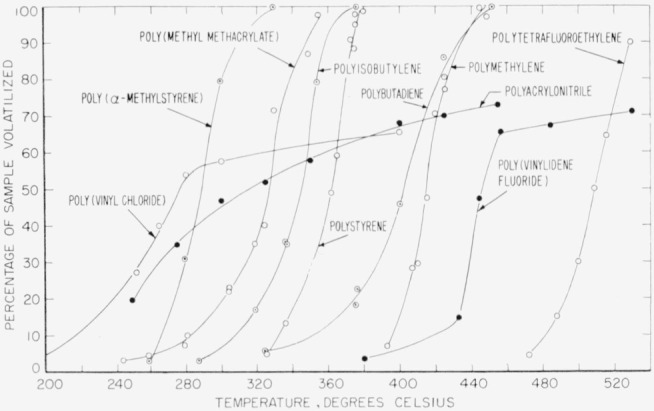
Relative thermal stability of polymers Curves were taken from the following references: poly(*α* methylstyrene) and poly(methyl methacrylate) [2]; poly isobutylene and polybutadiene [3]; polystyrene [2, 4]; polymethylene [5]; polytetrafluoroethylene and poly (vinylidene fluoride) [6]; poly (vinyl chloride) [7]; and polyacrylonitrile [8].

The thermal behavior of some representative members of the first two types of polymers is illustrated in figure 1. In this figure each point represents an experiment consisting of the pyrolysis in a vacuum of a polymer sample of about 50 mg or less during a 5-min preheating period, followed by 30 min of heating at the indicated temperature. Curves for polymers of type (1) are shown extending to about 100 percent volatilization, while curves for polymers of type (2) show stabilization at 60 to 70 percent volatilization.

As to polymers of type (3), polytrivinylbenzene serves as an example. This polymer, as well as its copolymers with styrene, have been studied by Winslow and coworkers [9, 10]. Polytrivinylbenzene begins to stabilize during pyrolysis at about 500° C and yields a carbonized residue of about 40 percent or less of the original sample, depending on the temperature and the rate of heating.

## 2. Materials Used

Polystyrene is the only polymer of group (1) used in the present investigation. This polymer is the same pure material that was used previously in pyrolysis studies [4]. It was prepared thermally and had a molecular weight of 230,000, as determined by the osmotic-pressure method.

The polymers of group (2) used in this investigation are poly(vinylidene fluoride), a white hard rubbery material, and polyacrylonitrile, in the form of a white powder. These materials are the same as those used previously in the work on pyrolysis [6, 8]. The poly(vinylidene fluoride) was polymerized by γ-radiation and would therefore be highly crosslinked and of high molecular weight. The polyacrylonitrile had a number-average molecular weight of 40,000.

Polytrivinylbenzene is the only polymer of group (3) used in this investigation. This polymer, in the form of a light-amber rod, was prepared thermally at 80° C in a nitrogen atmosphere, without reagents, and is the same material as that used by Winslow and his co-workers in their work [9, 10]^3^.

The work on these materials was carried out along two lines:

1. Pyrolysis at various temperatures, collection of volatile and nonvolatile products, and analysis of these products in the mass spectrometer and by a microcryoscopic method.
2. Measurement of rates of volatilization, and calculation of the activation energies of thermal degradation of the polymers involved.

## 3. Pyrolysis

Most of the experiments at temperatures below 500° C were carried out in an apparatus that has been described previously in connection with a study of pyrolysis in vacuum of cellulose and related materials [11] and also of tetrafluoroethylene and hydrofluoroethylene polymers [6]. The samples were placed in a small quartz tube that fits into a larger one projecting horizontally from the vacuum apparatus. The furnace was heated to the required temperature and was then moved quickly into position to heat the sample. It usually took about 5 min for the temperature of the sample in the small quartz tube to reach equilibrium temperature. The sample was then heated for 30 additional minutes at this temperature. For convenience, this apparatus will be referred to as apparatus I.

**Figure 2:**
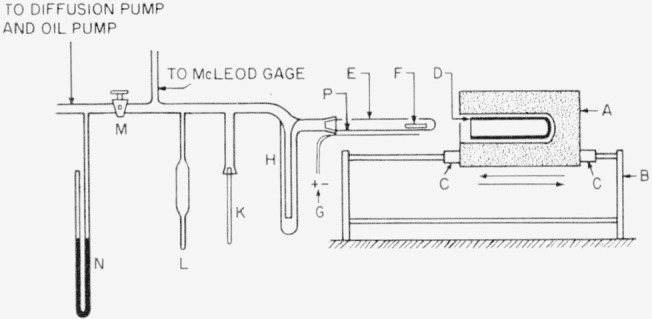
Apparatus for the pyrolysis of polymers at temperatures up to about 850° C A, electric furnace, resting on a steel frame B and provided with roller bearings C so that it can be moved quickly in a horizontal direction; D, stainless steel cylindrical cup serving as an efficient heat distributor; E, fused quartz tube connected to the glass vacuum apparatus by means of a ground joint; F, platinum cylindrical cup open at its left end (the sample is placed in this cup close to its bottom on the right end) ; G, platinum-platinum rhodium thermocouple held in a fine alundum tube and tied to the fused quartz tube E by means of platinum wire; H, liquid-nitrogen trap; K, Pyrex sample-tube for collecting products of pyrolysis volatile at room temperature, fraction V_25_; L, sample tube for collecting gaseous products volatile at the temperature of liquid nitrogen, fraction V_−190_; M, stopcock which is kept closed during pyrolysis and collection of fractions; N, manometer; P, point where V_pyr_ deposits.

Apparatus II, which was used in pyrolysis experiments at 500 to 850° C, is shown diagrammatically in figure 2. It is similar in many respects to apparatus I, except that it could be heated to a higher temperature, and the furnace could be moved more quickly in horizontal position by means of ball bearings. The sample holder was in the form of a cylindrical platinum crucible. A heavy stainless steel cylindrical cup fitting tightly into the furnace served as an efficient heat distributor for the quartz tube containing the platinum sample holder. Due to this efficient heat distribution the time required for the thermocouple G (fig. 2) to reach equilibrium after the furnace was moved into position for pyrolysis was only about 1 min for temperatures up to about 800° to 850° C. In some experiments apparatus II was used at temperatures below 500° C, as is described later.

In all the pyrolysis experiments described here, the vacuum was better than 10^−4^ mm of Hg, and the temperature control was about ±0.5° C. Temperature of the platinum cylinder was calibrated in a series of preliminary experiments against thermocouple G. Samples weighing about 10 to 50 mg were used.

### 3.1. Polystyrene

The thermal behavior of polystyrene at moderately high temperatures is well known. When heated in a vacuum [4] or in a neutral atmosphere [12] it slowly vaporizes at temperatures in the range of 250° to 400° C. In the present investigation the pattern of degradation was determined when a sample of this polymer, in a vacuum or in a neutral atmosphere is suddenly thrust into a hot zone at a temperature far above 400° C. Experiments were carried out at 362° and at 850° C in apparatus II, both in a vacuum and in helium at atmospheric pressure. As seen from table 1, a higher temperature or a higher pressure, or a combination of the two, produces a greater yield of small molecular fragments (V_25_).

Mass spectrometer analyses of fraction V_25_ for all 4 experiments arc given in table 2. Here again, as in the case of table 1, higher temperature and pressure favor a greater fragmentation of the volatile products. Fraction V_−190_ was not analyzed. The pressure developed in the apparatus due to this fraction was very low and, as was shown in previous work [4] below 500° C, it amounts to about 0.1 percent weight of the sample and consists mainly of CO.

Our results on the behavior of polystyrene when pyrolyzed at high temperature and pressure are in general agreement with those found in the literature for the same and other polymers. Midgley and Henne [13] pryolyzed natural crepe rubber in air at atmospheric pressure by heating it rapidly in an iron retort to 700° C and obtained a monomer yield of 10 percent of the pyrolyzed part, as compared with 5 percent [2] obtained in a vacuum in the temperature range 300° to 400° C. Bassett and Williams [14] heated smoked crepe rubber in air at a very fast rate to 600° C and obtained an isoprene yield of 19 percent. Boonstra and van Amerongen [15] pyrolyzed polyisoprene and other polymers in nitrogen at various pressures up to atmospheric, and at various temperatures up to 775° C. They obtained a maximum of 60 percent monomer from polyisoprene heated at 775° C and at 5 to 13 mm pressure. The monomer yield for polyisobutylene at 775° C and 15 mm pressure was 46 percent. For polybutadiene at 775° C and 5 mm pressure it was 14 percent. For polystyrene at 675° C and 15 mm pressure the monomer yield was 50 percent. The corresponding monomer yields at temperatures up to about 400° C and in a vacuum are 5 percent for polyisoprene, 20 percent for polyisobutylene, 1.5 percent for polybutadiene, and 40 percent for polystyrene [2].

A waxlike fraction V_pyr_ deposits inside of quartz tube E at point P (fig. 2), just to the left of the furnace, when the latter is in position for pyrolysis. It is subsequently collected by means of a suitable solvent or by scraping. This fraction from experiments 2, 3, and 4 was tested for average molecular weight by a microcryoscopic method [4] in benzene solution, with the following results:
*Experiment No.**Average molecular weight*231033104321Fraction V_pyr_ from polystyrene obtained in vacuum pyrolysis in the temperature range 325° to 375° C was found in previous work to have an average molecular weight of about 264. This fraction, according to information found in the literature and also in our earlier work, consists of a mixture of dimers, trimers, tetramers, and smaller amounts of longer chain fragments. The high molecular weight of V_pyr_ from experiments 2, 3, and 4 could indicate that they contain larger proportions of the heavier fragments than V_pyr_ from experiment 1.

### 3.2. Poly (Vinylidene Fluoride) and Polyacrylonitrile

As shown in figure 1, poly (vinylidene fluoride) and polyacrylonitrile tend to stabilize at higher temperatures, yielding a carbonaceous residue. Pyrolysis of these polymers has been studied previously [6, 8] at moderate temperatures, and now this study has been extended to 800° C (table 3).

Experiments 1 to 8 for poly (vinylidene fluoride) and 1 and 2 for polyacrylonitrile were carried out in apparatus I, and the other experiments in apparatus II. A comparison between slow heating and rapid heating to the same temperature is shown in experiments 9, 10, and 11 for poly (vinylidene fluoride).

Mass spectrometer analysis of fractions V_25_ and V_−190_ for those experiments in table 3 taken from references [6 and 8] have been described in those references. In the case of poly (vinylidene fluoride), V_25_ consisted mainly of HF. Fraction V_−190_ amounted to less than 0.1 percent of the total volatilized part and consisted mainly of H_2_ and CO. Fraction V_25_ from polyacrylonitrile consisted mainly of hydrogen cyanide, acrylonitrile, and vinylacetonitrile. The V_−190_ fraction was very small and consisted of H_2_. Analysis of fractions V_25_ from poly (vinylidene fluoride) from experiments 7, 9, 10, and 11 (table 3), confirmed previous results in that these fractions consisted of almost 100 percent HF. The V_−190_ fractions from the same experiments were too small to be analyzed. No attempt was made to analyze the V_25_ fraction from the polyacrylonitrile experiment at 800° C, in view of our earlier discovery [8] that constituents of this fraction tend to repolymerize on standing.

The average molecular weight of fraction V_pyr_ from polyacrylonitrile was found previously [8] to be 330. No suitable solvent could be found to make a microcryoscopic determination of the average molecular weight of V_pyr_ from poly(vinylidene fluoride). The residues from poly(vinylidene fluoride) were black masses which pulverized easily when pressure was applied; those from polyacrylonitrile were black powders.

### 3.3. Polytrivinylbenzene

Results of pyrolysis of this polymer are also shown in table 3. Stabilization begins at about 500° C, and the additional loss by volatilization from 500° to 800° C is only about 10 percent. These results are in fairly good agreement with those obtained by Winslow and co-workers [9, 10], whose method consisted in heating the samples slowly, the temperature rising at the rate of 100° C/hr. In Winslow’s work volatilization at corresponding temperatures is generally lower than in this work. On comparing experiment 6, in which the sample was heated slowly to 800° C over a period of 135 min and then kept at this temperature for 5 min, with experiments 7 and 8, in which the samples were thrust into the furnace at 800° C and kept there for 5 min, we find that the slower rate of heating resulted in smaller losses by volatilization.

Results of mass spectrometer analysis of fractions V_25_ from polytrivinylbenzene, in experiments 3, 4, and 8 (table 3), are shown in table 4; those for fractions V_−190_ for the same experiments, are shown in table 5. Here again, as in the case of polystyrene, a higher temperature of pyrolysis causes a greater fragmentation of the volatile products, as indicated by the relative yields of CH_4_ and H_2_ (table 5). Cryoscopic determinations in benzene solution of V_pyr_ from experiments 3, 5, and 6, showed average molecular weights of 372, 325, and 316 for the 3 fractions, respectively.

The carbonized residue from pyrolysis, even at 800° C, retained its original shape, but shrank in size and was hard and firm. Microchemical analyses of some residues, prepared for this purpose in a series of experiments, are shown in table 6. The fluorine in the poly(vinylidene fluoride) residues seems to cling tenaciously to the carbon, even at 800° pyrolysis. In the case of polyacrylonitrile, at 500° pyrolysis, the ratios C:H:N in the residue are not much different from those in the original material. This seems to support the assumption made by earlier investigators [16, 17, 18] with regard to the following possible structure of polyacrylonitrile residues from pyrolysis:

**Figure:**
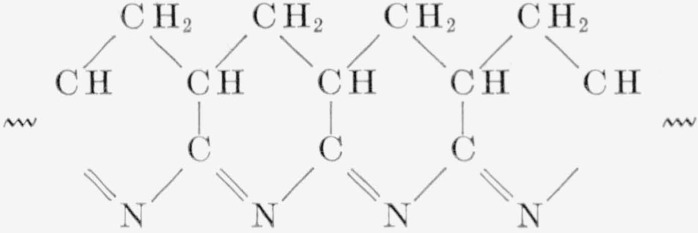


**Figure 3:**
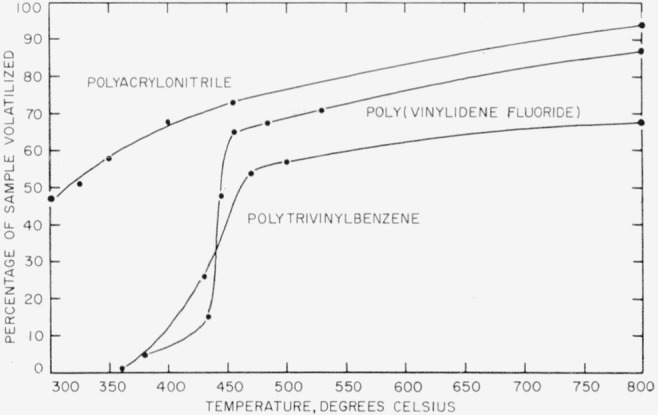
Relative thermal stability of high-temperature polymers.

The relative thermal stability of the 3 high-temperature polymers discussed in this paper is shown graphically in figure 3. The rate of volatilization goes up rapidly at about 450° C in the case of poly(vinylidene fluoride) and polytrivinylbenzene, while in the case of polyacrylonitrile there is a gradual decline of rate from 240° (fig. 1) to 800° C (fig. 3).

The average molecular weights of all fragments in the pyrolysis of each of the high-temperature polymers were calculated on the basis of mass spectrometer and cryoscopic analyses of the individual fractions. The results of these calculations are shown in table 7 for poly(vinylidene fluoride) and polytrivinylibenzene, for pyrolysis at 500° and 800° C. Calculations for polyacrylonitrile could not be made because of the uncertainty of mass spectrometer analysis of fractions V_25_ and V_−190_. With regard to the data shown in table 6, wherever the average molecular weight of V_pyr_ has not been determined, calculations were based on assumed values of either 300 or 500 for these weights, and the averages of the two results are shown in this table.

The significance of the overall average molecular weights of the volatile products of degradation lies in the fact that activation energies are expressed in units of energy per mole, the mole in this case being equal to the average molecular weight of the volatiles. This is true of poly(vinylidene fluoride), polyacrylonitrile, and polytrivinylbenzene, where the random scissions of the chain are not accompanied by unzipping reactions, as is the case, for example, with polystyrene or poly(methyl methacrylate). The uncertainty about secondary reactions discussed above is not involved here since the rate measurements were made in a vacuum and at low temperatures, where the composition of the volatiles is temperature independent.

## 4. Rates

**Figure 4:**
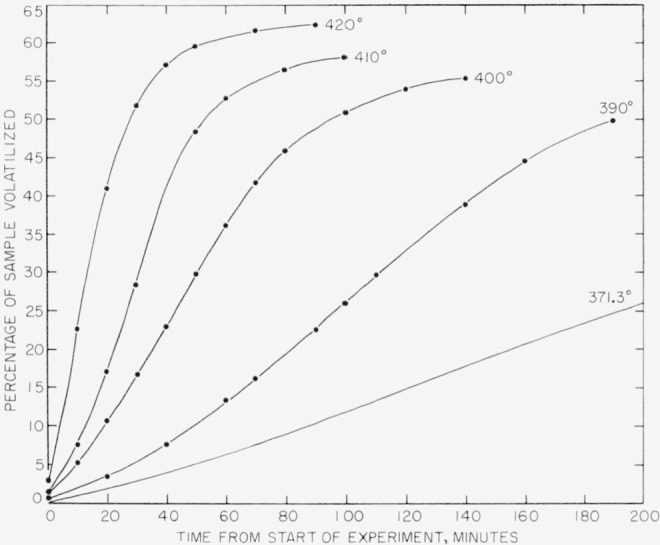
Thermal degradation of poly (vinylidene fluoride).

**Figure 5:**
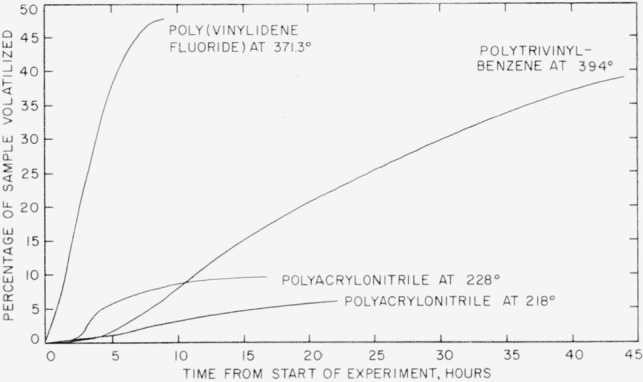
Thermal degradation of several polymers at slow rates.

**Figure 6:**
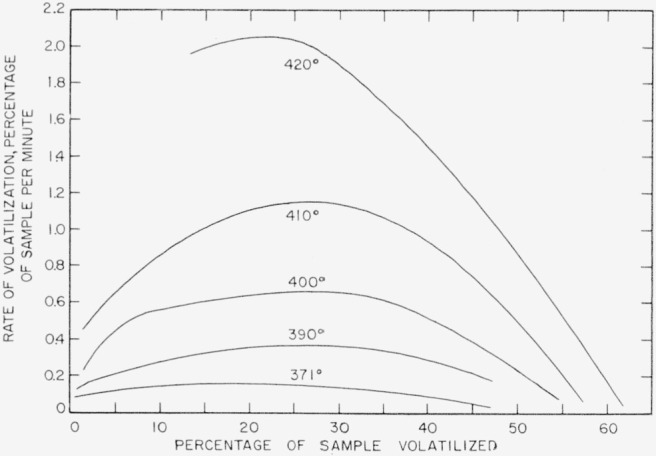
Rates of volatilization of poly(vinylidene fluoride).

**Figure 7:**
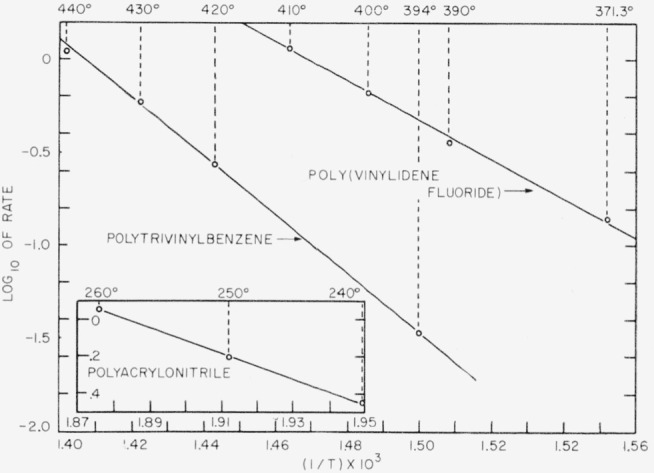
Rates of volatilization versus temperature of thermal degradation of polymers.

**Figure 8:**
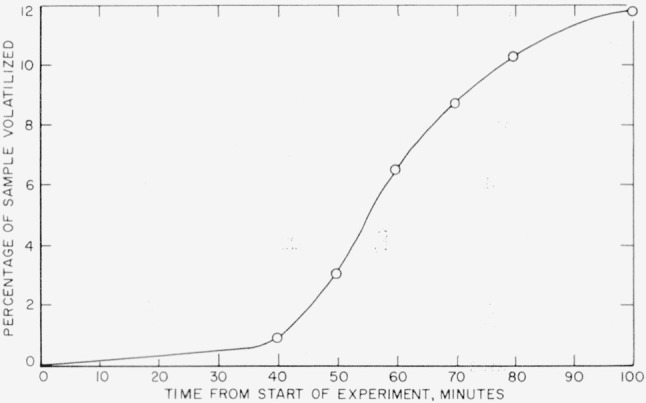
Thermal degradation of polyacrylonitrile at 240° C.

**Figure 9:**
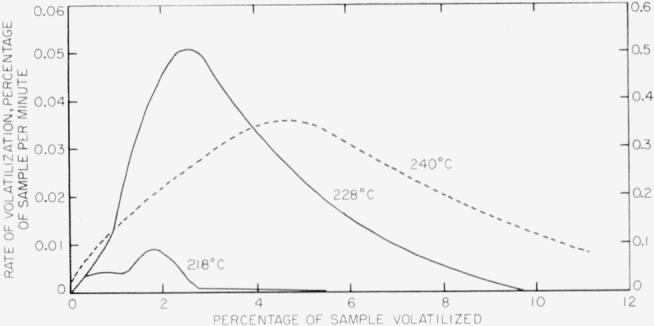
Rates of volatilization of polyacrylonitrile The rate-scale on the right side pertains to the 218° and 228° curves; the one on the left side pertains to the 240° curve.

**Figure 10:**
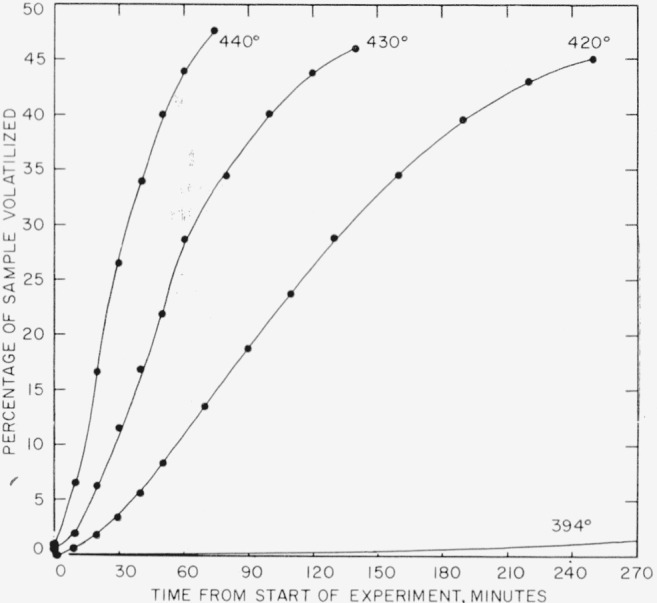
Thermal degradation of polytrivinylbenzene.

**Figure 11:**
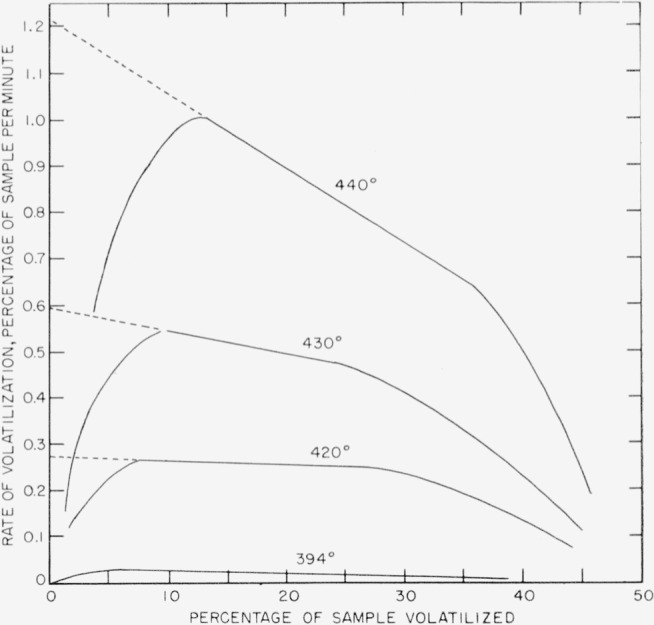
Rates of volatilization of polytrivinylbenzene.

Rates of thermal degradation were measured by the loss-of-weight method using two very sensitive balances enclosed in vacuum systems: a tungsten spring balance for the experiments involving faster rates at higher temperatures, and an electronic balance, provided with automatic recording of the loss of weight, for slower rates at lower temperatures. These balances and the experimental procedures have been described in detail in previous publications [19, 20]. Pertinent data on rate experiments for poly (vinylidene fluoride), polyacrylonitrile, and polytrivinylbenzene are given in table 8. The rate measurements and activation energies calculated from these rates using the Arrhenius equation, are shown graphically in figures 4 to 11 inclusive.

In figure 4 percentage volatilization of poly (vinylidene fluoride) is shown plotted versus time. Experiments at 390°, 400°, 410°, and 420° C were made in the spring balance. The experiment at 371.3° was made in the electronic balance. Only a small section of the 371.3° curve is shown in figure 4; the complete curve is shown in figure 5. The curves in figure 4 indicate volatilization losses up to about 3 percent at zero time. This is due to the fact that some volatilization of poly (vinylidene fluoride) in the higher temperature experiments took place during a 15 min heating up period, and the zero time was counted from the time when the temperature had reached equilibrium. The same conditions prevailed in the rate studies of the other two polymers, particularly those in the spring balance.

In experiments using the spring balance observations of loss of weight were made at regular intervals, and the points on the curves in figure 4, as well as in similar curves for the other polymers, represent these observations. In the experiments in the electronic balance, the weight losses were recorded continuously and automatically, and these losses are plotted accordingly in the 371.3° curve in figure 4 and in similar curves for other polymers.

Rates for poly(vinylidene fluoride) were calculated from the time-volatilization curves in figures 4 and 5, and are shown plotted in figure 6 versus percentage volatilization. The maximum rates, shown numerically in table 8 and plotted in figure 7, were used in calculating the activation energy. The activation energy thus obtained is 48 kcal/mole.

Results of rate measurements for polyacrylonitrile are shown plotted in figures 5 and 8. Experiments at 218° and 228° C (fig. 5) were made in the electronic balance; the experiment at 240° C (fig. 8) was made in the spring balance. Rates versus percentage volatilization in experiments at 218°, 228°, and 240° C are shown plotted in figure 9. The activation energy was calculated on the basis of maximum rates at 240° C, shown in figure 9, and at 250° and 260° C, in the spring balance described in our earlier work [8]. The numerical values of the maximum rates are given in table 8, and some of these are shown plotted in figure 7. The activation energy thus obtained was found to be 31 kcal/mole. Maximum rates at 218° and 228° C do not fit into this plot. It is quite likely that the reaction mechanism at lower temperatures differs from that at the higher temperatures.

Rate experiments for polytrivinylbenzene at 420°, 430°, and 440° C were carried out in the spring balance, and at 394° in the electronic balance. Volatilization-versus-time curves for these experiments are shown plotted in figure 10. Only a small section of the 394° curve is shown in figure 10. The whole curve is shown in figure 5. The rates-versus- percentage volatilization curves are shown plotted in figure 11. The activation energy can be calculated either on the basis of the extrapolated initial rates (shown as interrupted lines) in figure 11, also given numerically in table 8, or on the basis of maxima. The resulting activation energy is the same for both methods of calculation and amounts to 73 kcal/mole. The results plotted in figure 7 for polytrivinylbenzene are based on the extrapolated initial rates.

## 5. Summary and Conclusions

Polystyrene vaporizes completely, whether pyrolyzed slowly or rapidly, in a vacuum, or in a neutral gas at atmospheric pressure, in the temperature range 362° to 850° C. However, the chemical nature and relative amounts of the products of pyrolysis are functions of temperature and pressure. A higher temperature or a greater pressure, or a combination of the two, causes a greater fragmentation of the products. The same is true of polytrivinylbenzene with regard to temperature, which was the only variable studied. In the case of poly(vinylidene fluoride) most of the volatiles consist of HF, whether at low or high temperatures of pyrolysis. As to polyacrylonitrile, no definite conclusions can be drawn because of the highly unstable nature of the volatiles.

It is not clear from the present work whether the greater fragmentation at higher temperature and pressure takes place during the primary reaction when the fragments break off the polymer, or in a secondary reaction after the fragments have already been formed. At higher temperatures a fragment already formed might break up further while passing through the hot zone in the furnace. At higher pressures, a further breakup of a fragment could take place while its escape from the hot zone is retarded through collisions with other molecules.

Polytrivinylbenzene is highly crosslinked due to the trifunctional nature of the monomer. When heated at temperatures above 500° C it does not vaporize completely. Stabilization above 500° takes place quickly, resulting in a carbonaceous residue. In the case of poly(vinylidene fluoride) and polyacrylonitrile crosslinking most likely develops during pyrolysis, due to losses of H and F in one case and losses of H and CN in the other. The linking may occur between different sections of the same chains or between different chains. Additional stability is imparted to the chains by the formation of conjugated double bonds in the chains due to splitting off of HF or HCN.

The activation energy of the degradation reaction of polytrivinylbenzene is about the same as that for polymethylene. It is much higher than those of any of the other polymers shown in figure 1 except polytetrafluoroethylene. The high stability and high activation energy of polytrivinylbenzene are undoubtedly due to its highly crosslinked structure.

## Figures and Tables

**Table 1:** Pyrolysis of polystyrene in a vacuum and in helium at atmospheric pressure

Experiment No.	Condition	Temperature	Heating duration^a^	Volatilization	Fractions^b^, based on volatilization		
					V_pyr_	V_25_	V_−190_
							
		° *C*		%	%	%	%
1	vacuum	362	1 hr	83	57	43	Trace.
2	do	850	35 sec	100	32	68	Do.
3	helium	362	1 hr	83	45	55	Trace.^c^
4	do	850	35 sec	98	28	72	Do.^c^

a Time at operating temperature.

b Either some or all of the following fractions were collected in each pyrolysis experiment described in this paper:

(1) Residue; (2) fraction V_pyr_, volatile at the temperature of pyrolysis, but not at room temperature; (3) fraction V_25_, volatile at room temperature, but not at the temperature of liquid nitrogen; and (4) fraction V_-190_, volatile at the temperature of liquid nitrogen.

c Estimated.

**Table 2:** Mass spectrometer analysis of fraction V_25_ from polystyrene^a^

Component	Experiment 1	Experiment 2	Experiment 3	Experiment 4
				
	*Mole %*	*Mole %*	*Mole %*	*Mole %*
Styrene	94.4	34.0	50.5	12.9
Toluene	5.6	5.5	5.6	14.1
Benzene	0	58.2	43.9	18.5
Propadiene	0	1.9	0	1.1
Ethylene	0	0	0	49.2
Ethane	0	0	0	4.2
				
Total	100.0	100.0	100.0	100.0

a See table 1 for pertinent data on the 4 experiments.

**Table 3:** Pyrolysis of high-temperature polymers

Experiment	Heating duration	Temperature	Volatilization	Fractions, based on volatilization		
				V_pyr_	V_25_	V_−190_

POLY (VINYLIDENE FLUORIDE)						

	*min*	° C	*Percent*	*Percent*	*Percent*	*Percent*
1^a^	30	380	4	……….	……….	……….
2^a^	30	433	15	59	41	Trace.
3^a^	30	444	48	48	52	Do.
4	30	450	67	31	69	Do.
5^a^	30	456	66	……….	……….	……….
6^a^	30	484	68	……….	……….	……….
7	30	500	76	32	68	Trace.
8^a^	30	530	71	52	48	Do.
9	b140	800	68	27	73	Do.
10	5	800	82	39	61	Do.
11	5	800	87	41	59	Do.

POLYACRYLONITRILE						

1^c^	30	350	58	88	12	Trace.
2^c^	30	455	73	88	12	Do.
3	5	800	94	86	14	Do.

POLYTRIVINYLBENZENE						

1	30	360	0	……….	……….	……….
2	30	430	26	……….	43	……….
3	30	470	53	75	24	1
4	30	500	55	82	13	5
5	30	500	59	……….	8	……….
6	b140	800	63	……….	5	……….
7	5	800	68	……….	3	……….
8	5	800	68	84	5	11

a From reference [6].

b Sample was heated from room temperature to 800° C over a period of 135 min and then kept at this temperature for 5 min.

c From reference [8].

**Table 4:** Mass spectrometer analysis of fraction V_25_ from pyrolysis of polytrivinylbenzene

Component	Composition of V_25_, in mole percent of fraction		
	470° C (Expt. 3)	500° C (Expt. 4)	800° C (Expt. 8)
			
C_11_H_16_	……….	1.9	0.9
C_10_H_14_	0.2	6.4	3.6
C_9_H_12_	.5	9.1	8.8
C_9_H_10_	……….	3.0	2.0
C_8_H_10_	0.8	5.0	7.7
C_8_H_8_	……….	0.4	0.4
C_7_H_8_	0.5	1.1	4.9
C_6_H_6_	.2	0.3	2.1
C_6_H_10_	……….	.4	……….
C_5_H_10_	1.2	.6	0.4
C_5_H_8_	0.3	……….	……….
C_4_H_10_	1.8	……….	……….
C_4_H_8_	8.8	6.7	4.6
C_4_H_6_	0.3	……….	……….
C_3_H_8_	13.0	7.9	6.5
C_3_H_6_	18.0	9.9	8.0
C_3_H_4_	4.1	1.1	0.6
C_2_H_6_	34.5	21.1	19.9
C_2_H_4_	15.5	25.1	25.0
CH_4_	0.3	……….	4.6
Total	100.0	100.0	100.0

**Table 5:** Mass spectrometer analysis of fraction V_−190_ from pyrolysis of polytrivinylbenzene

Component	Composition of V_−190_, in mole percent of fraction		
	470° C (Expt. 3)	500° C (Expt. 4)	800° C (Expt. 8)
			
C_3_H_6_ and higher hydrocarbons	1.0	1.7	1.9
C_2_H_6_	……….	2.0	0.7
C_2_H_4_	……….	3.4	1.0
CH_4_	65.7	55.1	21.5
H_2_	33.3	37.8	74.9
Total	100.0	100.0	100.0

**Table 6:** Microchemical analysis of residues from pyrolysis of polymers

Polymer	Temperature	Residue	Analysis				
			C	H	O	F	N
							
Poly(vinylidene fluoride)	° *C*	%	a%	%	%	%	%
	……….	……….	37.5	a3.1	……….	a59.4	……….
	500	32.3	81.5	1.2	……….	17.3	……….
	800	17.0	88.9	1.3	……….	9.8	……….
Polyacrylonitrile	……….	……….	a68.0	a5.7	……….	……….	a26.4
	500	……….	75.1	4.0	……….	……….	20.9
Polytrivinylbenzene	……….	……….	a92.3	a7.7	……….	……….	……….
	500	43.6	94.9	5.1	……….	……….	……….
	800	31.5	98.6	1.4	……….	……….	……….

a Theoretical composition of original material.

**Table 7:** Average molecular weight of volatiles from pyrolysis of poly (vinylidene fluoride) and polytrivinylbenzene

Polymer	Average molecular weight	
	500° C	800° C
		
Poly (vinylidene fluoride)	29±0.2	29±0.2
Polytrivinylbenzene	128±8	53

**Table 8:** Rates of thermal degradation of poly (vinylidene fluoride), polyacrylonitrile, and polytrivinylbenzene

Temperture	Heating duration	Loss of weight	Rate

POLY (VINYLIDENE FLUORIDE)			

° C		%	*%/min*
371.3	9 hr	47.6	0.14 (max).
390	3 hr, 10 min	49.9	.36 Do.
400	2 hr, 20 min	55.5	.66 Do.
410	1 hr, 40 min	58.0	1.15 Do.
420	1 hr, 30 min	62.5	2.15 Do.

POLYACRYLONITRILE			

218	22 hr	6.0	0.01 (max).
228	16 hr, 50 min	9.7	.05 Do.
240	1 hr, 40 min	11.8	.36 Do.
a250	1 hr, 40 min	14.0	.63 Do.
a260	1 hr, 40 min	16.5	1.14 Do.

POLYTRIVINYLBENZENE			

394	44 hr	39.2	0.03 (initial)
420	3 hr, 30 min	45.2	.28 Do.
430	2 hr, 20 min	46.0	.59 Do.
440	1 hr, 20 min	47.8	1.22 Do.

a Data for 250° and 260° C were taken from our previous work [8].

## Footnotes

2 Figures in brackets indicate the literature references at the end of this paper.

3 The writers thank F. H. Winslow of Bell Telephone Co. for supplying them with the polytrivinylbenzene.
